# Real-time imaging of ipsilateral parathyroid glands by retrograde injection of methylene blue into the superior thyroid artery: a new intraoperative parathyroid protection method

**DOI:** 10.1186/s12893-024-02360-z

**Published:** 2024-04-13

**Authors:** Hanjie Guo, Yuxing Zhang, Saiyu Ren, Xiaodong Yang, Lei Tian, Yun Huang, Chaojun Zhang, Xiliang Zhang

**Affiliations:** 1 Department of General Surgery, The First Medical Center of PLA General Hospital, 28 Fuxing Road, Haidian, Beijing, 100853 People’s Republic of China; 2 School of Medicine, South China University of Technology, 382 Waihuan Rd E, Panyu, Guangzhou, 510006 People’s Republic of China; 3 Department of General Surgery, The Sixth Medical Center of PLA General Hospital, 6 Fucheng Road, Haidian, Beijing, 100048 People’s Republic of China

**Keywords:** Parathyroid glands, Methylene blue, Superior thyroid artery, Intraoperative parathyroid protection

## Abstract

**Background:**

Postoperative hypoparathyroidism caused by parathyroid injury is a problem faced by thyroid surgeons. The current technologies for parathyroid imaging all have some defects.

**Methods:**

Patients with differentiated thyroid carcinoma (DTC) who underwent unilateral thyroidectomy plus ipsilateral central lymph node dissection were recruited. We dissected the main trunk of the superior thyroid artery entering the thyroid gland and placed the venous indwelling tube into the artery. The sensitivity, specificity, accuracy, positive predictive value (PPV) and negative predictive value (NPV) were calculated.

**Results:**

A total of 132 patients enrolled in this single-arm clinical trial, 105 of them completed retrograde catheterization via the superior artery. The sensitivity was 69.23 and 83.33% respectively. The specificity was 72.91 and 64.89%. The accuracy was 72.91 and 64.89%. The PPV was 85.71 and 81.08%. The NPV was 22.58 and 45.45%. There were no patients with allergic reactions to the methylene blue, or methylene blue toxicity.

**Conclusions:**

Retrograde injection of methylene blue via the superior thyroid artery is an effective and safe method to visualize parathyroid glands. This method can accurately locate the target organ by ultraselecting the blood vessel and injecting the contrast agent while avoiding background contamination and reducing the amount of contrast agent.

**Trial registration:**

**Clinical trial registration numbers and date of registration:** ChiCTR2300077263、02/11/2023.

**Supplementary Information:**

The online version contains supplementary material available at 10.1186/s12893-024-02360-z.

## Introduction

With the advancement and wide application of imaging methods, thyroid cancer has become the solid malignant tumor with the fastest-growing incidence worldwide [1]. The prognosis of most differentiated thyroid cancers is good, and the 5-year survival rate from 2010 to 2016 was 98.3% [2]. Therefore, avoiding long-term surgical complications that would hinder normal postoperative life has become an important goal of thyroidectomy.

The main complications of thyroidectomy are recurrent laryngeal nerve and parathyroid injuries. At present, the clinical application of intraoperative neurophysiologic monitoring has played a positive role in the finding and functional protection of the recurrent laryngeal nerve during surgery [3, 4]. However, postoperative hypoparathyroidism caused by parathyroid injury is still a problem faced by thyroid surgeons [5]. The incidence of temporary and permanent hypoparathyroidism after thyroidectomy is 14–60% and 4–11%, respectively [6–13]. Some studies have reported the surgical experience of a surgeon, central lymph node dissection, and malignant tumors are risk factors for damaging the parathyroid glands during thyroidectomy [14–16].During radical thyroidectomy, thyroid specialists must rely on their experience to distinguish the parathyroid glands from many lymph nodes in the connective tissue on the dorsal side of the thyroid. When thyroidectomy is operated by high-volume surgeons (>100 thyroid procedures per year), the risk of postoperative parathyroid injury is significantly reduced [14]. Melot et al. reported that central lymph node dissection is the strongest independent risk factor for inadvertent parathyroidectomy during thyroidectomy [16].When thyroidectomy is combined with central lymph node dissection, the probability of unintended resection or ischemia of the parathyroid is significantly increased [6, 17, 18].As to whether malignant tumors are a risk factor for parathyroid injury, there is still controversy. Many studies report that malignant tumors are a risk factor for parathyroid injury [16]. But Melot et al. suggest that malignant tumors may be a confounding factor for inadvertent parathyroidectomy, as most malignant tumors require central lymph node dissection [16].

Although the current application of the nanocarbon-negative parathyroid imaging technology has improved the identification of parathyroid glands during surgery [19–24], this method does not directly visualize the parathyroid glands, and the imaging of the lymph nodes is determined by the injection site, lymphatic drainage pathway, and degree of lymph node fusion. The sensitivity and specificity of imaging vary greatly between studies and have not been given much focus [25, 26].

This study wanted to design a new method that could directly visualize the parathyroid glands during surgery. Because the superior parathyroid glands rely on the blood supply of superior thyroid artery and its anastomotic branches with inferior thyroid artery, while the anastomotic branches can also partially supply the inferior parathyroid glands. The study planned to retrogradely inject methylene blue through the superior thyroid artery during thyroidectomy to visualize the parathyroid glands to improve their recognition rate. The detective value of this method were evaluated by immune colloidal gold technique (ICGT) assay that detected parathyroid hormone (PTH).

## Materials and methods

### Patient selection

This single-arm clinical trial was approved by the institutional ethics committee of The Sixth Medical Center of PLA General Hospital. Between August 2020 and October 2021, 132 patients were enrolled from the Department of General Surgery of The Sixth Medical Center of PLA General Hospital. All patients were clearly diagnosed with DTC through fine-needle aspiration cytology before surgery and were assessed as stage T1-T2 by intraoperative measurement, which was consistent with indicators for unilateral thyroidectomy plus ipsilateral central lymph node dissection. Written consent was obtained from all participants. All patients underwent angiography with retrograde injection of methylene blue through the superior thyroid artery. Patients with hyperthyroidism or retrosternal goiter were excluded.

### Intraoperative visualization of parathyroid glands by methylene blue

**Fig. 1:**
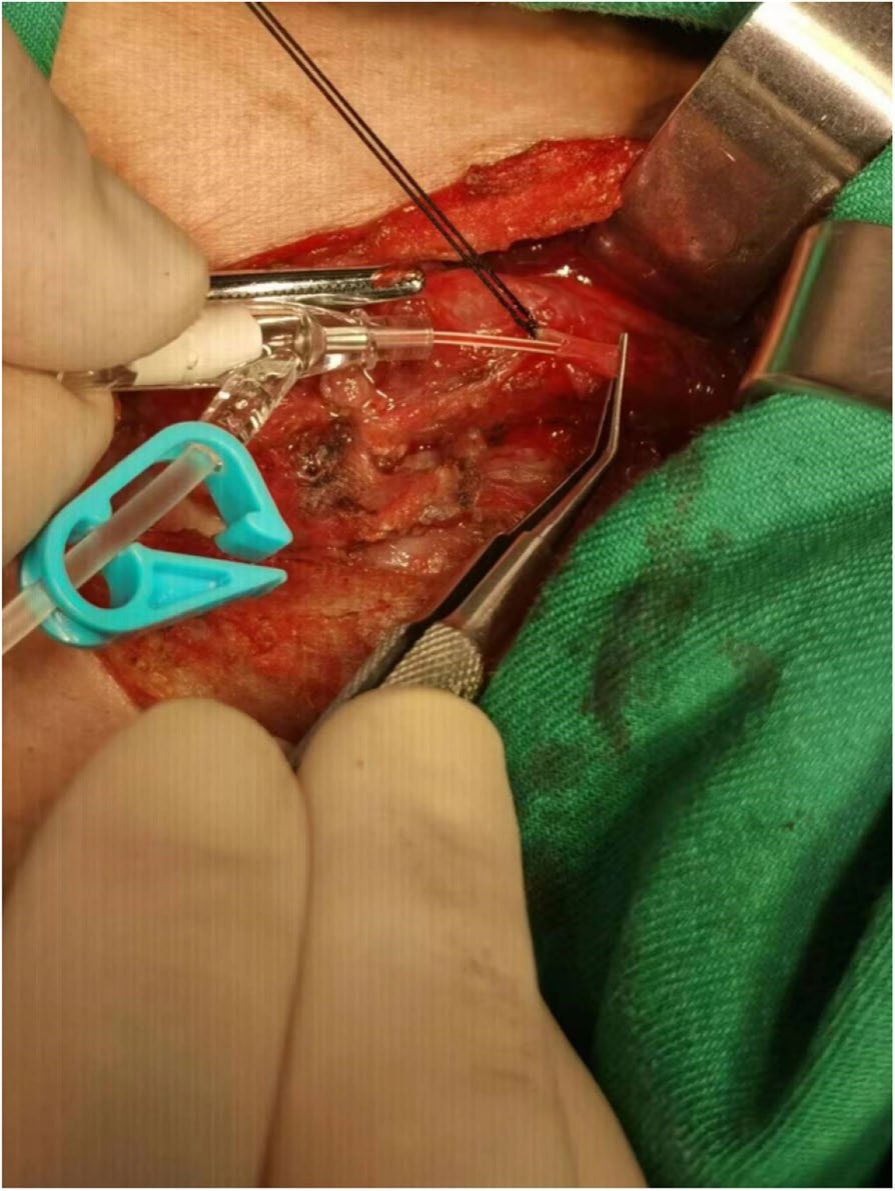
Puncture the anterior branch of superior thyroid artery in the direction of reverse flow

**Fig. 2:**
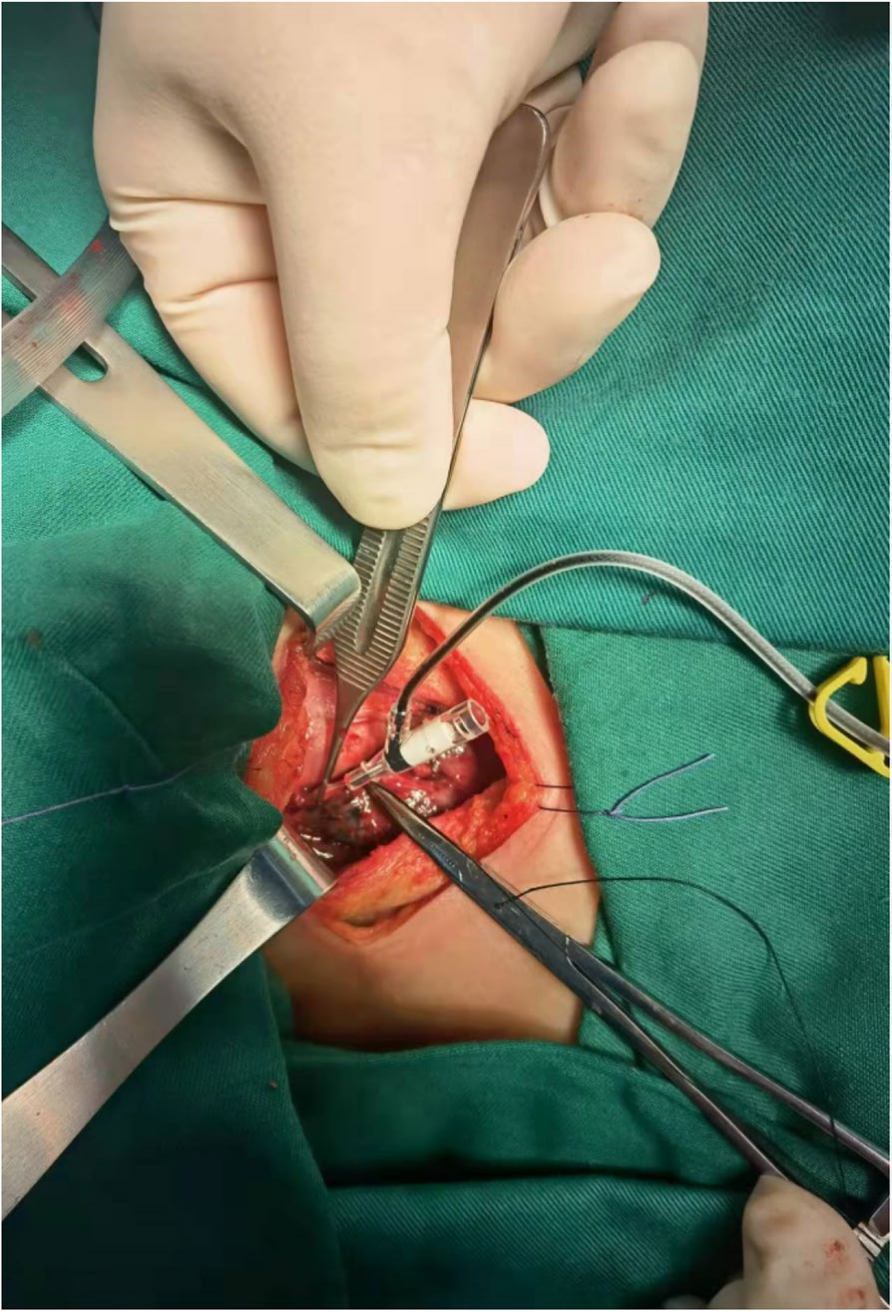
Retrograde injection of methylene blue into the superior thyroid artery

**Fig. 3:**
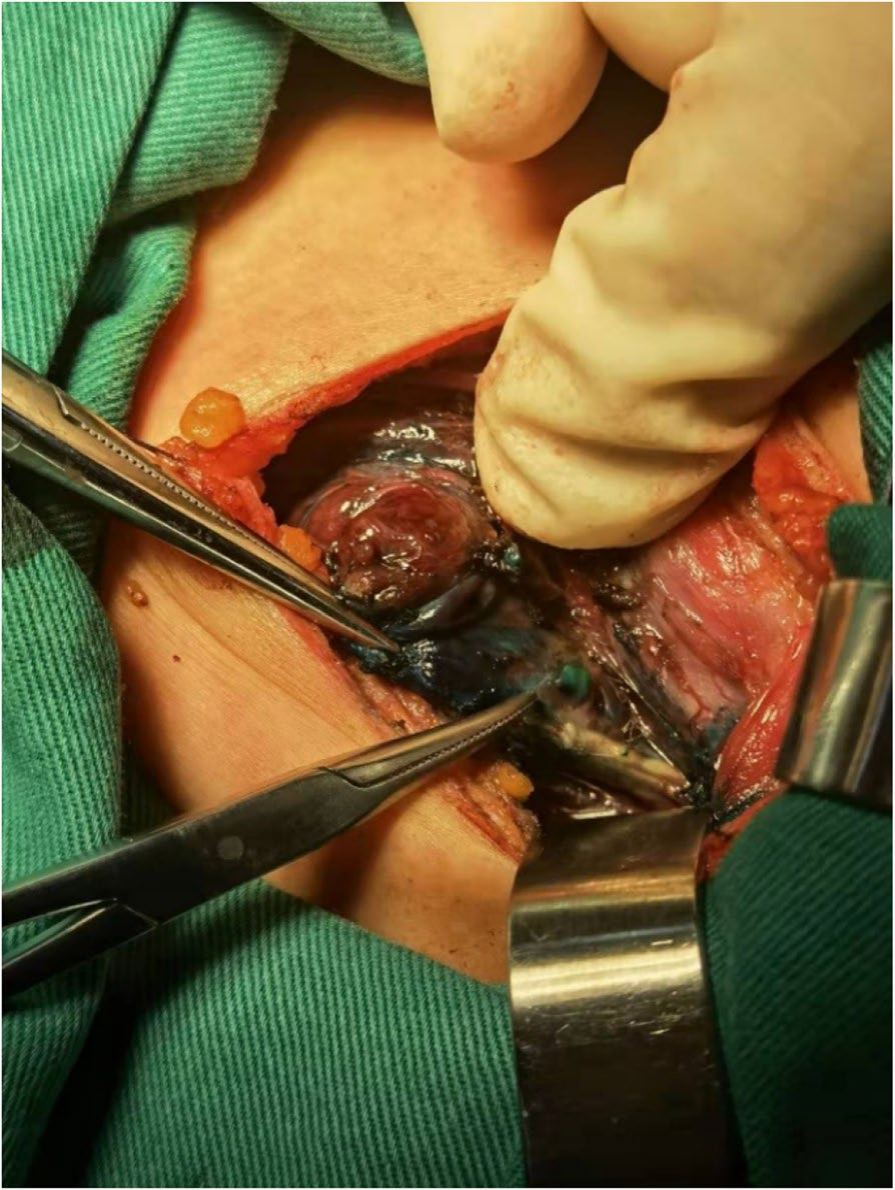
Intraoperative picture showing superior parathyroid glands after methylene blue dye

**Fig. 4:**
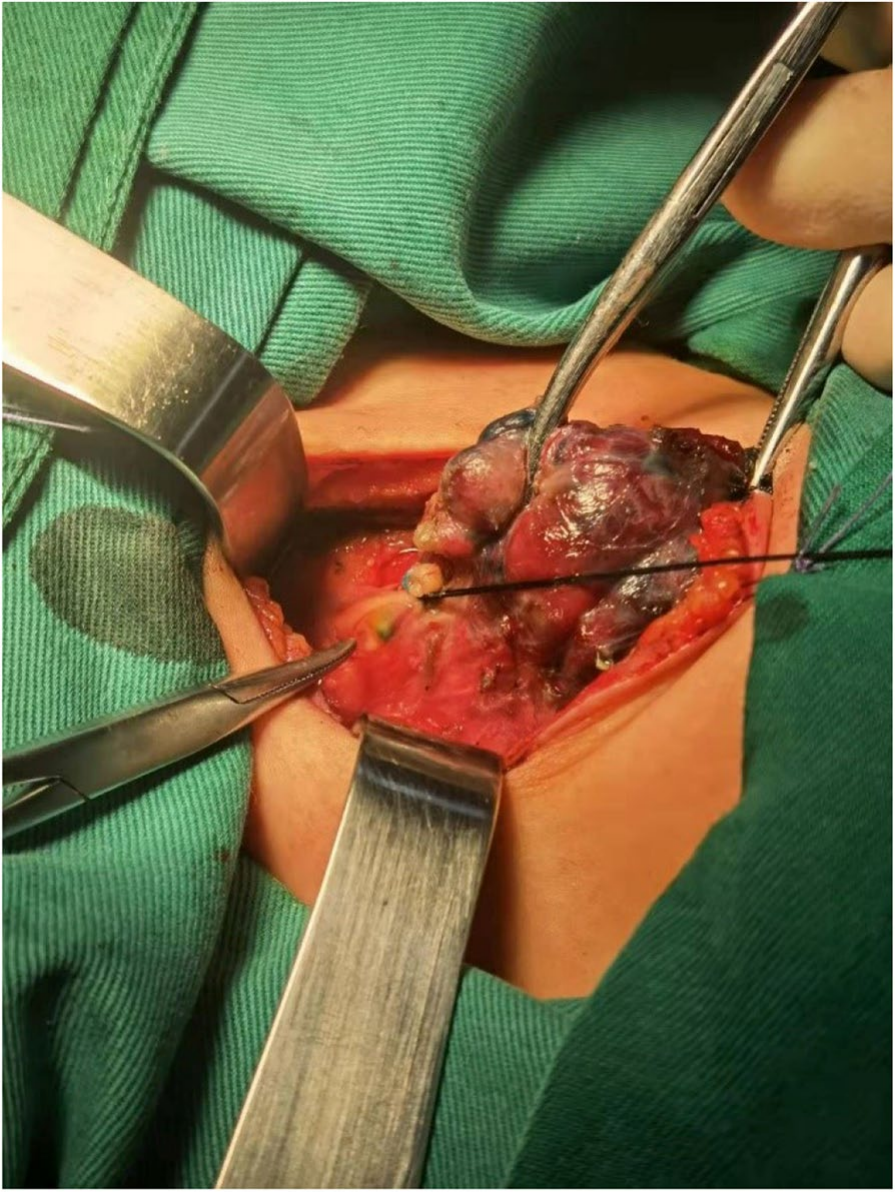
Intraoperative picture showing inferior parathyroid glands after methylene blue dye

The thyroid isthmus was dissected anteriorly to the trachea, the thyroid was detached from the cricothyroid muscle, and the lateral side of the thyroid was freed. The anterior branch of the superior thyroid artery entering the thyroid gland was dissected and ligated with a 4–0 thin suture. The artery was freed to the bifurcation of the anterior and posterior branches in the direction of reverse flow. The proximal end of the superior thyroid artery was clamped by microscopic tweezers to close the blood vessel to control the blood flow. Ophthalmic Venus scissors were used to cut the vessel wall transversely incision at the distal end of the vessel, and the venous indwelling tube (22Ga × 19 mm；Foshan Special Medical Co..ltd, Foshan, Guangdong, China) was placed into the artery in the direction of reverse flow (Figs. 1 and 2) without its front end entering the main thyroid artery. Methylene blue stock solution of 20 mg/2 ml was diluted to 5 mg/2 ml in saline, and 1 ml was slowly injected into the artery through the tube. The ipsilateral glandular lobe was completely excised while avoiding excision of the blue-stained tissue on the dorsal side of the glandular lobe (Figs. 3 and 4).

### Colloidal gold method to determine whether the blue-stained tissue is parathyroid

**Fig. 5:**
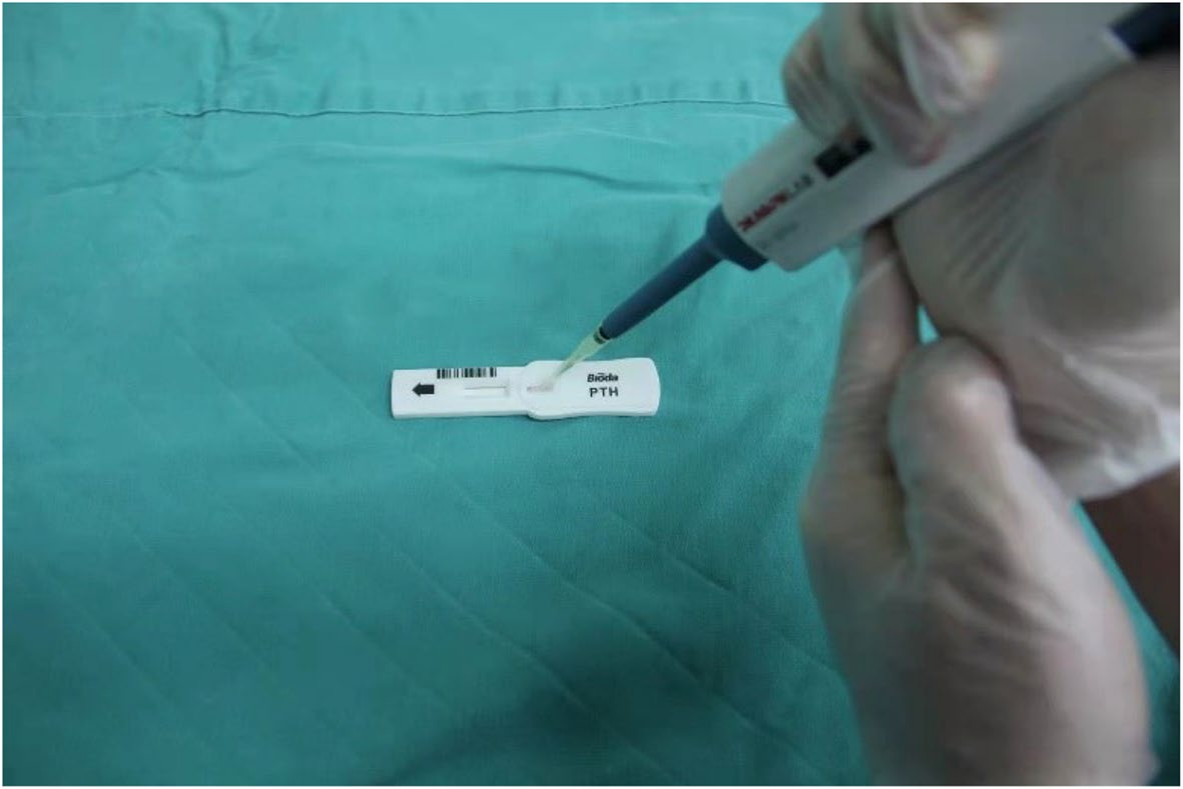
Eluent 90 μl was added dropwise to the sample loading area of the PTH-ICGT test strip and incubated for 10 min

**Fig. 6:**
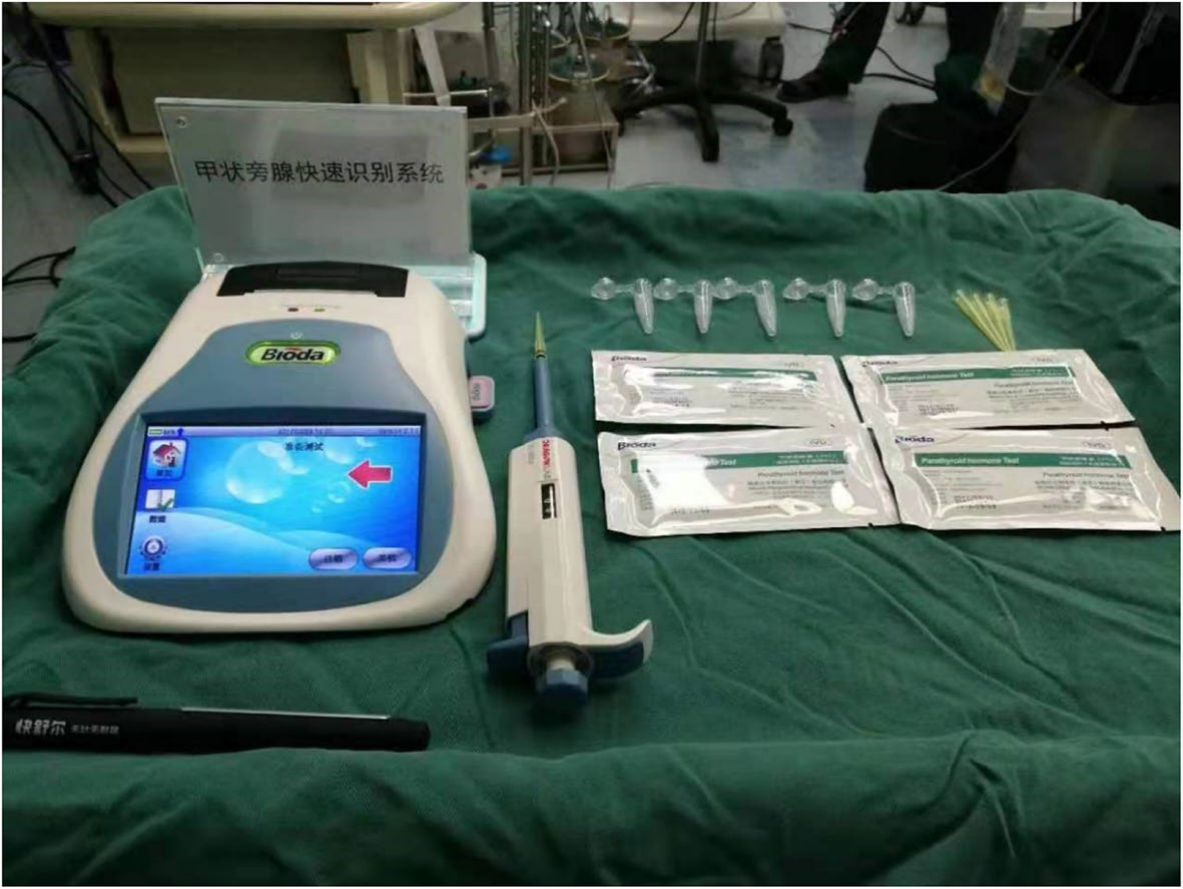
The strip loaded with reagent was placed in the test area to read the PTH results

The methylene blue on the dorsal side of the thyroid gland was observed again, and the levels of PTH in the blue-stained portion of the dorsal connective tissue of the thyroid lobe as well as in the non-blue-stained tissue that we identified as parathyroid tissue by the naked eye was quickly measured using a colloidal gold immunochromatographic assay (Bioda Diagnostics, Wuhan, Hubei, China) [6, 7]. The process was as follows: The target tissue was clamped with ophthalmic forceps three times and washed in 1 ml of normal saline, and the above action was repeated a total of three times. Eluent 90 μl was added dropwise to the sample loading area of the PTH-ICGT test strip and incubated for 10 min (Figs. 5). After the ICGT analyzer was turned on, it was set to the PTH routine test mode, and the strip loaded with reagent was placed in the test area to read the PTH results (Figs. 6). The same doctor added samples and operated the instrument for PTH detection. According to the instructions of the instrument, the cutoff value was set to 60.63 pg/ml. That is, if the detection value was higher than 60.63 pg/ml, the target tissue was deemed the parathyroid gland. The number of blue-stained tissues and the number and location of them determined by the ICGT were recorded; the number of unstained tissues that were determined to be parathyroid by the naked eye and the number and location of them that were determined to be parathyroid by the ICGT were recorded.

### Statistical analysis

SPSS 22.0 software was used for statistical analysis. The data are presented as the mean ± standard deviation. The two-independent-sample t-test was performed to compare two groups. The sensitivity, specificity, accuracy, positive predictive value (PPV), and negative predictive value (NPV) of each score were calculated.

## Results

**Fig. 7:**
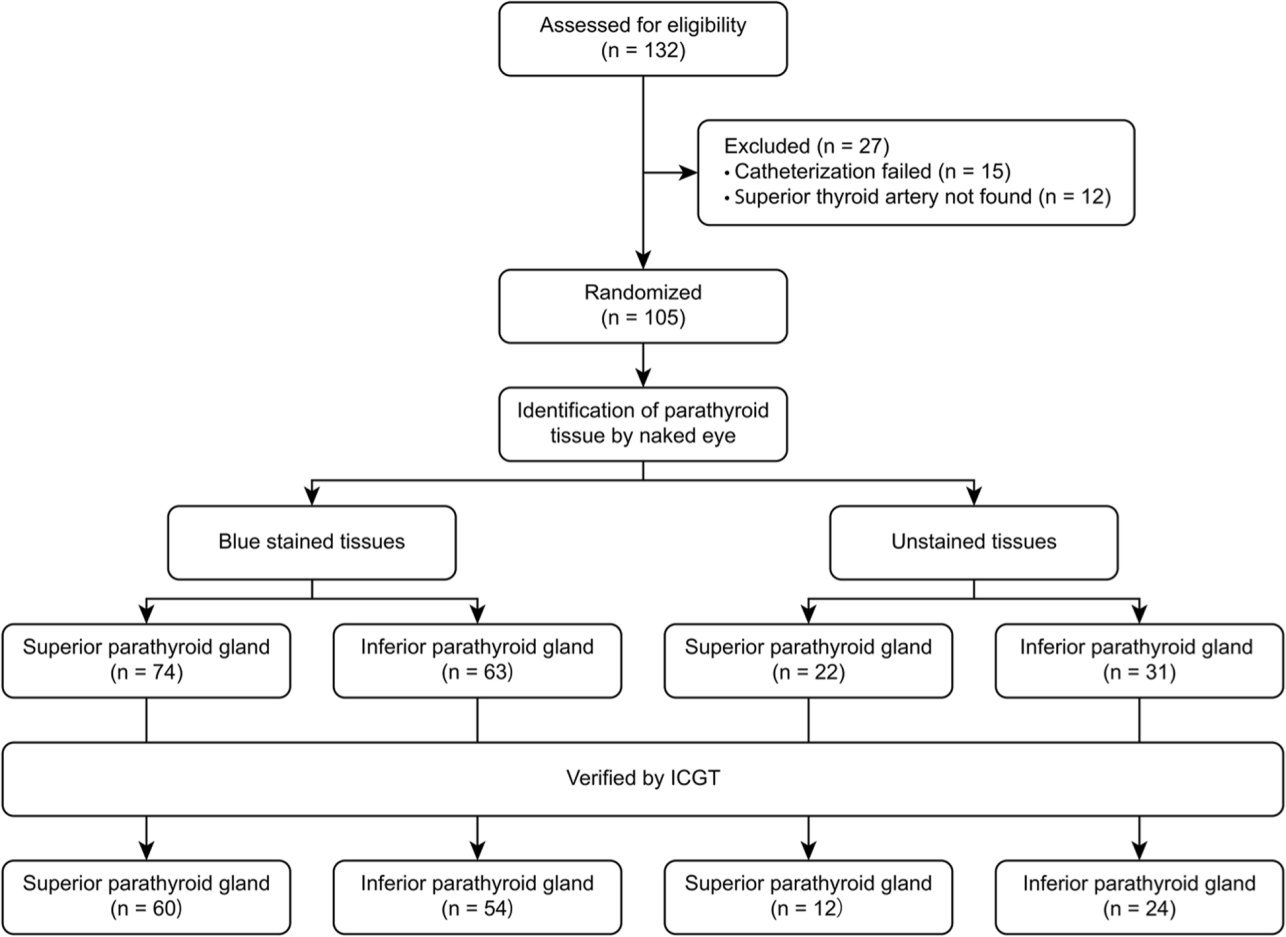
Clinical Research Flow Chart

Clinical Research Flow Chart (Fig. 7).

Among the 132 patients enrolled in the study, a total of 105 patients completed retrograde catheterization via the superior artery (Tables 1, 2, 3, 4 and 5). Among the other 27 patients, catheter placement failed in 15 (failure to insert or penetrate the vascular wall), and the main trunk of the superior thyroid artery was not found by intraoperative dissection in 12. Among the 105 patients who completed the catheterization, a total of 74 blue-stained tissues were identified as superior parathyroid glands by the naked eye, of which 60 were confirmed by the ICGT; 63 blue-stained tissues were identified as inferior parathyroid glands by the naked eye, of which 54 were confirmed by the ICGT. In addition, 22 unstained tissues were identified as superior parathyroid glands by the naked eye, of which 12 were verified by ICGT; 31 unstained tissues were identified as inferior parathyroid glands by the naked eye, 24 of them were confirmed by the ICGT.

**Table 1:** Patient characteristics

Characteristics		
Age (min–max)		48 (21–78)
Male		35 (26.5%)
Female		97 (73.5%)
Preoperative cytology	Papillary thyroid carcinoma (PTC)	119 (90.2%)
	Follicular thyroid carcinoma (FTC)	13 (9.8%)
	With nodular goiter	85 (64.4%)
	With Hashimoto disease	22 (16.7%)
Size of thyroid nodule (cm)		0.93 ± 0.59

**Table 2:** Comparison of PTH and blood calcium before and after surgery

	Preoperation (1 day before)	Postoperation (2 days after)	*P* value
Parathyroid hormone (pg/ml)	56.32 ± 25.86	37.80 ± 26.42	< 0.001
Calcium ion (mmol/L)	2.32 ± 0.09	2.14 ± 0.13	< 0.001

**Table 3:** Identification of the superior parathyroid glands by the naked eye after intraoperative retrograde angiography confirmed by ICGT

Identification by the naked eye	ICGT-confirmed results		Total
	Superior parathyroid gland	Not Superior parathyroid gland	
Local blue staining	60	14	74
No local blue staining	12	10	22
Total	72	24	96

**Table 4:** Identification of the inferior parathyroid glands by the naked eye after intraoperative retrograde angiography confirmed by ICGT

Identification by the naked eye	ICGT-confirmed results		Total
	Inferior parathyroid gland	Not inferior parathyroid gland	
Local blue staining	54	9	63
No local blue staining	24	7	31
Total	78	16	94

**Table 5:** The value of intraoperative retrograde angiography through the superior thyroid artery for the intraoperative identification of parathyroid glands

Identification value	The locally blue-stained site was the superior parathyroid gland (location)	The locally blue-stained site was inferior parathyroid gland (location)
	(96)	(94)
Sensitivity	83.33% (60/72)	69.23% (54/78)
Specificity	41.66% (10/24)	43.75% (7/16)
Accuracy	72.91% (70/96)	64.89% (61/94)
Positive predictive value	81.08% (60/74)	85.71% (54/63)
Negative predictive value	45.45% (10/22)	22.58% (7/31)

## Discussion

During thyroidectomy, manipulation, devascularization, or unintended resection of a normal parathyroid gland might result in a period of hypoparathyroidism and associated hypocalcemia [5]. A prospective clinical trial by Viola et al. [6] showed that after 5 years of follow-up, a higher prevalence of permanent hypoparathyroidism was observed in the total thyroidectomy plus central lymph node dissection group than in total thyroidectomy gland (19.4% versus 8.0%, *P* = 0.02) [6].

Although some classical studies [27] have proposed identifying parathyroid glands, adipose tissue, and lymph nodes based on size, weight, color, shape, and consistency, it remains challenging to accurately identify 3–4 parathyroid glands during total thyroidectomy. The method of identifying parathyroid glands by weight in physiological saline must be done after the removal of parathyroid glands, and ultimately, parathyroid glands can only be transplanted ectopically. In fact, in South Korea, where the incidence of thyroid cancer is relatively high, despite surgeons having extensive experience, the difficulty in visually identifying parathyroid glands during surgery results in a probability of post-thyroidectomy hypoparathyroidism as high as 6–10% [28].

Intraoperative imaging techniques can effectively improve the identification rate and protection rate of parathyroid glands. For example, intravenous injection of methylene blue imaging technology, autologous fluorescence imaging technology, Intraoperative indocyanine green angiography,and nano-carbon negative imaging technology. Although these four techniques are effective methods for intraoperative identification of parathyroid glands, their application also has corresponding limitations.

1. Intravenous injection of methylene blue imaging technology: The earliest report on intraoperative parathyroid imaging was reported by Dudley NE in 1971 [29], in which parathyroid glands were located by intravenous injection of methylene blue during neck dissection. However, a systematic review of intravenous injection of methylene blue in parathyroid surgery by Patel et al. [30] indicated that methylene blue is efficacious for identifying enlarged parathyroid glands but poor at visualizing normal parathyroid glands.
2. Autologous fluorescence imaging technology: Paras et al. [31] were the first to report the autofluorescent properties of parathyroid glands. In the near-infrared (NIR) spectrum [31], Benmiloud et al. [32] demonstrated in a before-and-after control study that thyroidectomies using NIR imaging for parathyroid glands preservation were associated with a significantly lower rate of postoperative hypocalcemia and a reduced rate of parathyroid glands. However, this imaging method requires the purchase of a NIR imaging instrument, and the external light source needs to be turned off during parathyroid imaging, affecting the continuity of the surgery.
3. Intraoperative indocyanine green angiography: The study by Parfentiev et al. [33] reported that intraoperative indocyanine green vascular imaging with near-infrared fluorescence is indeed a safe and reproducible technique. However, similar to autologous fluorescence imaging technology, intraoperative indocyanine green angiography requires specialized imaging equipment and fluorescence detection systems, making its cost relatively high and not suitable for all medical environments. Additionally, indocyanine green is light-sensitive, requiring the closure of external light sources during parathyroid imaging, affecting the continuity of the surgery.
4. Nano-carbon negative imaging technology: In 2012, Chinese scholars began to use nanocarbons to image the central lymph nodes in thyroidectomy, and they believe that this is helpful for the imaging of the parathyroid [19–24]. The meta-analysis of Wang et al. [25] showed that carbon nanoparticles can improve the extent of neck dissection and preserve the normal anatomic structure and physiological function of the parathyroid. However, this method also requires the purchase of specific imaging equipment, and its imaging is mainly visualizing lymph nodes, not parathyroid glands. It still requires the identification of parathyroid glands in adipose tissue by the naked eye. This method has only been reported by mainland Chinese teams, and there is still some controversy about it elsewhere [26].The use of ICGT instead of nano-carbon for parathyroid imaging faces similar challenges [34].

This study aimed to overcome the shortcomings of the abovementioned parathyroid imaging methods. We chose retrograde injection of methylene blue through the inferior thyroid vein to image the parathyroid glands. This method allows for the visual observation of parathyroid imaging with the naked eye. It enables the removal of glandular lobes or the dissection of central lymph nodes while observing the blue-stained parathyroid glands, avoiding inadvertent cutting of the parathyroid glands. The implementation of this method is based on the characteristics of the blood supply of the parathyroid glands is closely related to the posterior branch of superior thyroid artery and its anastomotic branches with inferior thyroid artery [35]. The blood supply of the superior parathyroid glands is mainly from the posterior branch of the superior thyroid artery, while the inferior parathyroid glands is from the inferior thyroid artery. Due to the abundant anastomotic branches of the superior and inferior thyroid artery [36], methylene blue can be retrograde injected into the anterior branch of superior thyroid artery to develop the superior parathyroid glands through the posterior branch and develop the inferior parathyroid glands through the anastomotic branches.

The ICGT had high sensitivity and accuracy in imaging parathyroid glands, but its specificity was low. This may have been related to the variation in the blood supply of the parathyroid glands, or it may be due to the close proximity of some parathyroid glands to the thyroid. It may be related to the position of catheterization, for example, if the front end of the tube be punctured into the main trunk of the superior thyroid artery, the tube will obstruct the origin of the posterior branch, methylene blue may not enter the posterior branch and fail to develop the parathyroid glands.

We also tried retrograde injection of methylene blue into the inferior thyroid artery to contrast the parathyroid glands. However, due to the anatomical variation and narrow diameter of the inferior thyroid artery, the success rate of catheterization is low. Even if the puncture is successful, the development rate of the inferior parathyroid glands is also low, for some parathyroid glands are close to the thyroid and the puncture point is actually located at the proximal end of the parathyroid glands. The methylene blue spray technique, which sprays methylene blue on the thyroid bed to take advantage of the property that the parathyroid glands can rapidly absorb methylene blue, causing local discoloration. The parathyroid glands are localized by the local discoloration properties of the area [37].

In this study, there were no complications, such as phototoxicity [38], serotonin syndrome [39], or hyperpyrexia [40], that may occur with intravenous injection of methylene blue, and no patient’s urine was tinted blue after the operation. This may be related to the injection dose. Usually, the dose of peripheral intravenous injection of methylene blue to visualize the parathyroid glands is 7.5 mg/kg, while the total dose of methylene blue injection in this study was 2.5 mg. By comparing the PTH levels and blood calcium before vs. after surgery, we found that although they were significantly lower 2 days after surgery, they were still within the reference ranges because the enrolled patients underwent unilateral thyroidectomy. We did not have long-term follow-up data of PTH or blood calcium.

If the patients had Hashimoto’s disease, the puncture success rate was low. Among the 105 patients who completed the puncture, 13 patients had Hashimoto’s disease, while the 9 of 27 patients who did not complete the puncture had Hashimoto’s disease. Among the 13 patients with Hashimoto’s disease who underwent puncture successfully, a total of six blue-stained tissues were identified as superior parathyroid glands by the naked eye, of which five were confirmed by the ICGT; six blue-stained tissues were identified as inferior parathyroid glands, four of which were confirmed by the ICGT. The data showed that if the patient had Hashimoto’s disease, the puncture success rate was significantly lower, and even if the puncture was successful, the rate of parathyroid blue staining was also significantly lower than that of the patients without Hashimoto’s disease. A possible explanation for these results is the dense inflammatory process that surrounds the thyroid gland, which makes visual identification and preservation of parathyroid blood supply more complicated [41].

Our study has several limitations. It was a single-arm experiment without a control group, and only patients undergoing unilateral thyroidectomy were included. The effect of this method on the blood calcium level and the PTH level after bilateral thyroidectomy was not evaluated. We assessed the parathyroid hormone levels and blood calcium concentrations in all enrolled patients one day before surgery and on the second day postoperatively. We found that even in cases where only one lobe of the thyroid was excised, regardless of the successful identification of the ipsilateral two parathyroid glands during the procedure, there was a significant reduction in parathyroid hormone levels and blood calcium concentrations on the second day postoperatively compared to the day before surgery. This may be attributed to the inevitable reduction in blood supply to the parathyroid glands after ligating the superior and inferior thyroid arteries, before the establishment of collateral circulation, leading to a compromised output pathway of the parathyroid glands. We obtained follow-up data on parathyroid hormone levels and blood calcium concentrations at approximately two months postoperatively for about half of the enrolled patients. We found no significant differences in parathyroid hormone levels and blood calcium concentrations at two months postoperatively compared to the preoperative values. This could be related to the reconstruction of local collateral circulation in the surgical area, and it is also possible that compensatory increases in parathyroid hormone release from the contralateral parathyroid glands occurred. Due to the lack of prior implementation of this technique, our primary objective was to initially verify its safety, specifically assessing the presence of significant adverse reactions during and after the procedure. Subsequently, we aimed to evaluate its imaging effectiveness, confirming whether the highlighted areas corresponded to the parathyroid glands. Once satisfactory safety and imaging effectiveness were established, we plan to conduct further research to investigate whether this technique can significantly reduce the incidence of refractory hypocalcemia following total thyroidectomy. In mainland China, there has been widespread implementation of early screening for thyroid cancer this year. Most patients are diagnosed with small tumors, and often, only unilateral lobectomy along with ipsilateral central lymph node dissection is required. We aim to recruit a substantial number of patients in a short timeframe to validate the safety and imaging effectiveness of this technique. Therefore, the inclusion criteria have been set to include only those requiring unilateral thyroid lobectomy along with ipsilateral lymph node dissection. Although some advantages of our novel imaging method seem obvious, a prospective randomized study is needed to gain more reliable information, and we are planning to conduct such a study in the future.

## Conclusions

We present an effective and safe method to visualize parathyroid glands by retrograde injection of methylene blue via the superior thyroid artery. This method can accurately locate the target organ by ultraselecting the blood vessel and injecting the contrast agent while avoiding background contamination and reducing the amount of contrast agent. Although further studies assessing the feasibility and validation of this technique are needed, retrograde injection of methylene blue via the superior thyroid artery should be considered whenever the parathyroid glands need to be explored and protected.

### Supplementary Information

**Supplementary material 1.**

**Supplementary material 2.**

## Data Availability

The datasets generated and/or analyzed during the current study are not publicly available due to the nature of patients with Thyroid cancer but are available from the corresponding author on reasonable request.
