# Supplementary material for: Real-time imaging of ipsilateral parathyroid glands by retrograde injection of methylene blue into the superior thyroid artery: a new intraoperative parathyroid protection method
Source: BMC Surg. 2024 Apr 13;24:108. doi: 10.1186/s12893-024-02360-z (PMC11015547; doi:10.1186/s12893-024-02360-z)
Supplement: Supplementary file 1 — Supplementary material 1. [file 12893_2024_2360_MOESM1_ESM.docx]

Informed consent

Applicant (researcher) : Zhang Xiliang

Project name: Clinical study of intraoperative superselective injection of methylene blue for imaging parathyroid glands

Study protocol version number and date: Ver= 1.003/17/2020

Informed Consent version number and date: Ver= 1.003/17/2020

Application department: General Surgery, Sixth Medical Center, Chinese People's Liberation Army General Hospital

**Dear___________(Sir/Madam)，**

You will be invited to participate in a clinical study of a scientific research topic/new technology and new business. The following items describe the background, purpose, method, benefits and possible risks or inconveniences brought to you during the research process, as well as your rights and interests, etc. Please be sure to read carefully before participating in the clinical study. The information provided in this informed consent form can help you decide whether to participate in the clinical study. If you have any questions, please ask the applicant (investigator) in charge of the study to ensure that you fully understand the relevant content. Your participation in this study is voluntary. If you agree to participate in this clinical study, please sign the informed consent statement.

**Study name and Purpose:**

**Name：**Clinical study of intraoperative superselective injection of methylene blue for imaging parathyroid glands

**Purpose：**To evaluate the efficacy and safety of intraoperative methylene blue injection for parathyroid protection in open total thyroidectomy

**Research background (difference from current clinical routine diagnosis and treatment methods) :**

Thyroid cancer is the fastest growing solid tumor in the world, and surgery is the most effective treatment for most thyroid cancers. Due to the special location and character of the parathyroid gland, it is easy to be damaged during thyroidectomy or central lymph node dissection, resulting in postoperative hypoparathyroidism. The incidence of temporary and permanent hypoparathyroidism after thyroid surgery is reported to be 14-60% and 4%-11%, respectively. At present, the mainstream intraoperative parathyroid development technology is negative carbon nanoparticle development, but this technology has not been widely promoted because it does not directly develop the parathyroid, which is easy to pollute the operating field and affect the operation. We improved the intraoperative imaging technique of parathyroid gland by dissecting the superior thyroid artery during the operation and injecting methylene blue retrograde through the blood vessel to develop the parathyroid gland. This method conforms to the anatomical structure, is simple in operation, does not require the purchase of large medical equipment, and can develop accurately without increasing the medical costs of patients. The effectiveness and safety of this surgical method will be verified in this study.

**Research methods and contents:**

**I. Inclusion and exclusion criteria:**

Inclusion criteria: ① differentiated thyroid cancer was confirmed by preoperative FNAC (fine needle puncture pathology) or intraoperative freezing pathology; ② According to the ATA (American thyroid society) standard, for only exists in the unilateral glands, the largest nodule diameter less than 10mm (intraoperative frozen pathology clear) of papillary carcinoma defined as tiny papillary carcinoma, unilateral gland lobe plus gorge resection, unilateral central area lymph node dissection, for the rest of the differentiated cancer with total thyroidectomy, bilateral central area lymph node dissection.

Exclusion criteria: ① age less than 15 years old or more than 75 years old; ② pregnant women; ③ there are serious organic diseases need to complete surgery as soon as possible; ④ History of surgery in the neck area; ⑤ in line with the diagnosis of retrosternal thyroid; ⑥ FNAC or intraoperative pathologic consideration of medullary carcinoma or undifferentiated carcinoma.

**II. Research steps:**

A total of 300 patients with differentiated thyroid cancer treated in our hospital were randomly divided into three groups with 100 patients in each group. Groupe A was the experimental group, which underwent superselective vascular methylene blue injection imaging; Groupe B positive control group: negative development by nano carbon injection; Groupe C negative control group: Intraoperative area imaging was not performed.

Groupe A: Before the unilateral thyroid gland lobe was removed, the main trunk of the superior thyroid artery was dissected to enter the thyroid gland, and the blood vessels were ligation with 4-0 thin threads. The blood vessels were dissected to the rear of the common carotid artery on the same side in the direction of reverse blood flow, and the superior thyroid artery was clipped at the intersection of the common carotid artery with microvessels to control the proximal and distal blood flow. The blood vessel wall was cut transversally with ophthalmic Venus scissors, and the hose was placed into the artery in the direction of reverse blood flow. 20mg/2ml methylene blue was diluted into 5mg/2ml with normal saline and injected into the artery slowly (2min) through a hose. After the lateral glandular lobe was completely removed (avoiding the removal of the parachromatic gland), the development of the dorsal thyroid methylene blue was observed again. The level of parathyroid hormone (PTH) in the predicted parathyroid tissue was rapidly determined by the immune colloidal gold technique (ICGT) to determine whether the prediction was correct. For bilateral lobectomy, contralateral imaging and parathyroid identification were performed in the same way.

Groupe B: The surface of unilateral thyroid gland lobe was fully exposed, carbon nanoparticle suspension injection was extracted with a 1 ml skin test syringe and slowly injected 0.2 mL around tumor tissue (upper and lower), and withdrawn before injection to avoid blood vessel injection. The level of parathyroid hormone (PTH) in the predicted parathyroid tissue was also rapidly determined by the immune colloidal gold technique (ICGT) after complete resection of the lateral lobe to determine whether the prediction was correct. For bilateral lobectomy, contralateral negative imaging and parathyroid identification were performed with the same method.

Groupe C: Unilateral or bilateral lobotomy is routinely performed. Attention should be paid to the protection of the parathyroid gland according to experience and surgical skills. After complete resection of one side of the gland lobe, the immune colloidal gold technique(ICGT) to determine the level of parathyroid hormone (PTH) in the naked parathyroid tissue to determine whether the prediction is correct; The contralateral parathyroid was determined by the same method.

**III. Observation indicators and follow-up plan:**

1. The number of correct parathyroid judgments and the total judgments of Groupe A/B/C were counted;

2. For patients with bilateral thyroidectomy and isthmus and central lymph node dissection, intravenous blood sampling was performed to check parathyroid level and blood calcium level before and on the first morning after surgery;

Follow-up plan: (1 follow-up for all enrolled patients 6 months after surgery)

1. If the level of parathyroid hormone in the blood was reviewed 6 months after surgery, if it was less than 50% of the preoperative value, and the patient still had hypocalcemia symptoms such as numbness, tingling, and hand-foot convulsion, it was defined as permanent hypoparathyroidism. The number of patients in Groupe A/B/C group meeting the diagnostic criteria for permanent hypoparathyroidism was counted.

**Research process and duration:**

This clinical study was conducted in three stages, namely, screening period (days -7-0), perioperative period (day and day after surgery), and follow-up period (6 months after surgery).

**Funding sources for research and possible conflicts of interest:**

The funds of the clinical research institute involving the developer and related follow-up data collection are provided by the Beijing Municipal Science and Technology Commission "Capital Clinical Diagnosis and treatment Technology Research and Demonstration Application Special Fund". All researchers declare no conflict of interest with this project.

**Possible benefits for subjects:**

In this trial, you will have the possibility to undergo intraoperative superselective intravascular injection of methylene blue for parathyroid imaging during thyroid surgery, which is safe and effective without increasing intraoperative injury or extending postoperative recovery time. If properly used, this technique can protect parathyroid blood supply and avoid parathyroid misincision, thus reducing the possibility of postoperative hypocalcemia and improving the postoperative quality of life of patients.

**Subject's possible risks and discomfort:**

For the experimental group, methylene blue was injected into the inferior thyroid artery during the operation, such as excessive injection, which could cause dizziness, malignity, vomiting, chest distress, abdominal pain and other symptoms; If the injection dose is too large, in addition to aggravating the above symptoms, there are also headaches, reduced blood pressure, increased heart rate with arrhythmia, heavy sweating and consciousness disorders, blue urine after medication, and tingling of the urethral opening when urinating. In order to avoid the above symptoms, the surgical operators of the project team will strictly control the total dose (<10mg) when injecting methylene blue, and reduce the dose of methylene blue into the systemic circulation by temporarily blocking the proximal end of the inferior thyroid artery.

**Treatment and financial compensation for subjects' research-related injuries:**

For this clinical study, the sponsor will not purchase commercial insurance for the included subjects. However, the sponsor undertakes to bear the cost of treatment and the corresponding financial compensation for the subjects who have suffered injuries related to the clinical trial, except for the damage caused by the fault of the medical institution and its medical staff during the diagnosis and treatment activities.

**Subjects may be assigned to study groups:**

You will be randomly assigned to receive the treatment or control group. You and your doctor in charge do not know in advance which type of surgery you are undergoing, but no matter which type of surgery you are undergoing, it is in line with the current medical practice and will not cause any delay in the condition or the probability of postoperative complications.

**Alternatives to this study:**

For patients who require unilateral or bilateral thyroidectomy, the following treatment options are available in addition to open surgery.

1. Complete endoscopic thyroid surgery. Without incision in the neck, surgery through the chest wall, axillary, and areolar approaches requires the establishment of a long subcutaneous tunnel and intraoperative injection of large amounts of CO2 to form an artificial air neck, which may lead to subcutaneous emphysema or even mediastinal emphysema.
2. Endoscopic assisted thyroidectomy. Only a small incision is made in front of the neck, and the operation is completed under the combination of direct vision and endoscopy, without the need to establish an artificial air neck. It's a minimally invasive procedure.
3. Radio frequency ablation. For some patients who are considered to have benign thyroid nodules before surgery, radiofrequency ablation may be used, which has no incisions, is less invasive, and does not require hospitalization. However, pathological specimens could not be obtained, and there was also the possibility of accidental injury to recurrent laryngeal nerve and part of parathyroid gland.

Depending on your underlying condition and the severity of your condition, your doctor will recommend the best treatment for you.

**Confidentiality of medical records:**

By signing the informed consent, you consent to the researcher and his or her staff collecting personal data about you for use in the study (" Study Data "), including your date of birth, gender, identity card, home address, photographs taken, and personal data about your physical or mental health.

All medical records and research materials that identify you will be kept confidential to the extent permitted by law. However, researchers, sponsors and representatives, monitors (people responsible for checking how research is conducted and ensuring that information is collected correctly) and, in certain circumstances, regulatory bodies and ethics committees will inspect and copy confidential information that can identify you. All personal information in the study will be processed in accordance with national and local data protection laws.

You have the right to request the researcher and sponsor to protect your research data and other relevant information. You also have the right to correct any inaccuracies in these data. If you make a request, please contact your doctor first, he/she can help you contact the sponsor if necessary. If you withdraw your informed consent, the investigator will no longer use your data or disclose it to others, and the sponsor may still use the study data obtained before you withdrew your informed consent.

The results of this study may be published in medical journals and presented at medical conferences. You will not be identified in any of these publications.

**Free treatment programs and other related benefits that may be available during the study period:**

In this clinical study, patients will try 1-2 boxes of "parathyroid hormone (PTH) test kit" free of charge in the scope of intraoperative resection, and the cost will be borne by the Department responsible for "Beijing Science and Technology Plan project - Capital Clinical diagnosis and treatment technology Research and Demonstration Application".

For this clinical study, the sponsor will sign a written agreement with the funder (Beijing Science and Technology Commission), which will specify the financial situation and payment method of this clinical trial in detail. For details, please refer to the agreement signed by the parties.

**Voluntary study participation and withdrawal:**

You may choose not to participate in the study, or withdraw from the study at any time after notifying the investigator without discrimination or retaliation, and any of your medical treatment and benefits will not be affected.

If you require additional diagnosis/treatment, or if you do not comply with the study protocol, or for any other reasonable reason, the investigator may terminate your continued participation in the study.

You can keep abreast of information and research progress related to this study. If you have questions related to this study, or if you have any discomfort or injury during the study, or if you have questions about the rights and interests of participants in this study, You can contact

(the name of the researcher or person involved in the study) at (phone number).

If you need to know about the rights and interests of participants in this study during the study, you can contact the Drug Clinical Trial Ethics Committee Office of the Sixth Medical Center of the PLA General Hospital (formerly Naval General Hospital) at 010-66957608.

**Subject declaration**

I have read this Informed consent carefully, I have had the opportunity to ask questions and all questions have been answered. I understand that participation in the study is voluntary and that I may choose not to participate in the study or withdraw at any time after notifying the investigator without discrimination or retaliation, and that any of my medical benefits and entitlements will not be affected.

If I require additional diagnosis/treatment, or if I do not comply with the study protocol, or if there are other reasonable reasons, the investigator may terminate my continued participation in the clinical study.

I voluntarily agree to participate in the clinical study, and I will receive a signed copy of the Informed Consent form.

Subject's signature: Date:

If the subject is unable to sign the informed consent due to incapacity or other reasons, or the subject is a minor, the subject's guardian shall sign it.

Signature of Guardian: Date:

Relationship with subjects:

Reasons why subjects cannot sign informed consent:

**Investigator's statement**

I have accurately informed the subjects of the informed consent and answered their questions, and the subjects voluntarily participated in this clinical study.

Investigator Signature: Date:
