# Supplementary material for: Real-time imaging of ipsilateral parathyroid glands by retrograde injection of methylene blue into the superior thyroid artery: a new intraoperative parathyroid protection method
Source: BMC Surg. 2024 Apr 13;24:108. doi: 10.1186/s12893-024-02360-z (PMC11015547; doi:10.1186/s12893-024-02360-z)
Supplement: Supplementary file 2 — Supplementary material 2. [file 12893_2024_2360_MOESM2_ESM.docx]

### **Project summary**

### **Basic principle:** Intraoperative imaging of parathyroid glands can effectively improve the identification rate and protection rate of parathyroid glands. However, both positive and negative imaging techniques commonly used in clinical practice have shortcomings. Compared with the traditional positive and negative development methods, super selected vascular methylene blue injection development enables surgeons to better identify the location and blood supply of the parathyroid gland during thyroid surgery by improving the intraoperative parathyroid development technology, reducing the intraoperative parathyroid injury, reducing the incidence of postoperative hypoparathyroidism, and improving the quality of life of patients after thyroid surgery.

### **Objective:** This study aims to reduce the probability of parathyroid injury during thyroid surgery and reduce the proportion of postoperative hypoparathyroidism patients by improving intraoperative parathyroid imaging technology.

### **Methods:** The superior thyroid artery was dissected and contrast agent was injected into the thyroid by reverse puncture. The parathyroid gland was explored according to the suggestion of contrast agent development. A total of 300 patients with differentiated thyroid cancer admitted to the Sixth Medical Center of the PLA General Hospital in March 2020 were randomly divided into three groups, with 100 cases in each group. They were divided into group A: The experimental group received ultra-selective vascular methylene blue injection imaging; Group B positive control group: negative development of nano carbon injection; Group C negative control group: intraoperative area imaging was not performed.

### **General information**

### **Protocol title：**Clinical study of intraoperative superselective injection of methylene blue for imaging parathyroid glands

### **Protocol identifying number and date：**Ver=1.0 03/17/2020

### **Name and address of the funder：**Beijing Science and Technology Project. Building 1, No. 57, Canal East Street, Tongzhou District, Beijing

### **Name and title of the study leader：**Xiliang Zhang，High title.

### **The address and telephone numberof the research site：**The Sixth Medical Center of PLA General Hospital，010-66951426.

### **Rationale & background information**

### Thyroid cancer is the fastest growing solid malignancy worldwide. In the cancer ranking spectrum of various countries and regions, thyroid cancer has been on the rise in the past decade, and its incidence rate ranks first among women^[1]^. Despite the increasing incidence, thyroid cancer has a good prognosis. According to the SEER system statistics of the National Institutes of Health (NIH), the mortality rate in 2016 was 0.5/100,000, and the 5-year survival rate from 2009 to 2015 was 98.2%^[2]^.

### The good prognosis of thyroid cancer is due to the normalization of treatment. For differentiated thyroid cancer, which accounts for the vast majority of thyroid cancer, surgical resection is currently the preferred treatment, among which total thyroidectomy and central lymph node dissection have become the most commonly used surgical treatment for differentiated thyroid cancer ^[3]^. Due to the good prognosis of thyroid cancer, most patients can return to normal work and life, but the serious complications caused by surgery can last a lifetime, which brings great inconvenience to patients' work and life.

### The main complications of thyroidectomy are recurrent laryngeal nerve and parathyroid gland injury. At present, the clinical application of intraoperative neuroelectrophysiological monitor (IONM) has played a positive role in the search and functional protection of recurrent laryngeal nerve during the operation ^[4,5]^. However, postoperative hypoparathyroid function caused by parathyroid injury is still a difficult problem for thyroid surgeons ^[6]^. Both direct injury (excessive operation, burning or unintentional excision) and indirect injury (impaired blood supply) may lead to postoperative hypoparathyroidism. It has been reported in literature that the incidence of temporary and permanent hypoparathyroidism after thyroid surgery is 14-60% and 4%-11%, respectively ^[3,7-11]^. Moreover, bilateral central lymph node dissection may increase the risk of hypoparathyroidism ^[12,13]^.

### Transient hypoparathyroidism may cause transient hypocalcemia, but it will not affect the quality of life of patients. However, permanent hypoparathyroidism can cause permanent low calcium symptoms, mostly manifested by hand and foot numbness and limb convulsions, which seriously affect the quality of life of patients and become the main factor in medical disputes. Therefore, the protection of parathyroid glands has become an urgent problem to be solved in total thyroidectomy and central lymph node dissection.

Intraoperative imaging technology can effectively improve the parathyroid identification rate and protection rate. At present, the commonly used imaging techniques in clinic are:

(1) Positive development: Positive development of the parathyroid gland refers to the use of developer to stain the parathyroid gland to facilitate accurate identification of the parathyroid gland during surgery. The most commonly reported positive parathyroid developer is methylene blue. Since Dudley used intravenous methylene blue to locate the parathyroid gland during neck dissection in 1971 ^[14]^, a large number of clinical reports have reported that peripheral intravenous methylene blue injection before surgery can show the parathyroid gland during surgery ^[15]^. Other experiments have shown that methylene blue is light in color and difficult to distinguish by naked eye. After 2013, clinical studies using fluorescence exploration equipment combined with methylene blue injection to develop parathyroid glands appeared ^[16-18]^. However, the above clinical studies also show the limitations of methylene blue development: 1. Pathological parathyroid tissue is easily stained by methylene blue, while normal parathyroid tissue has a low staining rate and poor effect; 2. In these clinical trials, the dose of intravenous methylene blue was relatively large (0.4-0.5mg/kg), and neurotoxic adverse events occurred in some patients after surgery, so the safety could not be guaranteed; 3. Fluorescence imaging equipment investment is huge, only suitable for clinical research, it is difficult to carry out routine in lower-level hospitals. More importantly, due to the small number of participants and defects in the experimental design, there is still a lack of high-level experimental evidence to confirm that intraoperative injection of methylene blue parathyroid imaging can indeed reduce postoperative hypocalcemia.

(2) Negative development: that is, nano carbon parathyroid negative development identification and protection technology. Nano carbon suspension injection (referred to as nano carbon) is a suspension made of nano carbon particles with a particle diameter of 150 nm and a high lymphatic system tendency ^[19]^. Because the capillary endothelial cell gap is 20-50 nm, while the capillary lymphatic endothelial cell gap is 120-500 nm, and the basal membrane is underdeveloped, carbon nanoparticles injected into the thyroid tissue will not enter the blood vessels, but can quickly enter the lymphatic vessels or enter the lymphatic vessels after phagocytosis by macrophages, and then remain and gather in the lymph nodes. Thus, the lymph nodes of the thyroid gland and its drainage area are black stained ^[20]^. Since most of the parathyroid glands are located in the central region and do not accept lymphatic return from the thyroid gland, most of the lymphatic vessels and lymph nodes in the thyroid gland and its drainage area will be black stained after injection of carbon nanoparticles into the thyroid tissue, while the parathyroid glands will not be black stained, making them easily distinguished-from the black-stained thyroid gland and lymph nodes. Compared with developers such as methylene blue, carbon nanocrystalline has the characteristics of strong lymphatic tendency, fast tracing speed, high black staining rate, long duration and high color contrast with surrounding tissues. Some clinical studies have shown that the use of negative carbon nanography technology can significantly reduce the incidence of postoperative hypocalcemia ^[21-24]^. Despite the obvious advantages compared with methylene blue, the negative development technology of carbon nanoparticles still has the following shortcomings: 1. In theory, carbon nanoparticles only stain thyroid and lymph node tissues, and the parathyroid glands still cannot be distinguished from fat droplets, thymus and other tissues; 2. Extravasation of the needle eye contaminate the surgical field, increasing the difficulty of surgery; 3. When the tumor is too large or the lymphatic system is invaded by the tumor, the injection and imaging effects are not good; 4. The above defects limit the further popularization of negative development technology of carbon nanoparticles.Moreover, the searchable literature shows that the current clinical studies of nano-carbon parathyroid negative imaging are all made by the Chinese mainland team, indicating that this technology still lacks international recognition.

We improved the technique of intraoperative imaging of parathyroid glands by dissecting the superior thyroid artery during the operation and injecting methylene blue retrograde through the blood vessel to develop parathyroid glands. This method conforms to the anatomical structure, is simple in operation, does not require the purchase of large medical equipment, has accurate imaging, and does not increase the medical costs of patients.

### **References**

[1] Davies L, Welch HG. Current thyroid cancer trends in the United States[J]. [JAMA Otolaryngol Head Neck Surg.](http://p2.ytwgk.cn/pubmed/24557566) 2014, 140(4): 317-322.

[2] SEER cancer statistic review, 1975-2016. <https://seer.cancer.gov/statfacts/html/thyro.html>

[3] Haugen BR, Alexander EK, Bible KC, et al. 2015 American Thyroid Association Management Guidelines for Adult Patients with

Thyroid Nodules and Differentiated Thyroid Cancer: The American Thyroid Association Guidelines Task Force on Thyroid Nodules and Differentiated Thyroid Cancer[J]. Thyroid. 2016, 26(1): 1-133.

[4] Dralle H, Sekulla C, Haerting J, et al. [Risk factors of paralysis and functional outcome after recurrent laryngeal nerve monitoring in thyroid surgery[J].](https://www.ncbi.nlm.nih.gov/pubmed/15657592) Surgery. 2004, 136(6): 1310-1322.

[5] Musholt TJ, Clerici T, Dralle H, et al. [German Association of Endocrine Surgeons practice guidelines for the surgical treatment of benign thyroid disease[J].](https://www.ncbi.nlm.nih.gov/pubmed/21424798) Langenbecks Arch Surg. 2011, 396(5): 639-649.

[6] Thyroid Surgeons Committee, Surgeons Branch, Chinese Medical Doctor Association. Expert consensus on parathyroid protection during thyroid surgery[J]. Chinese Journal of Practical Surgery. 2015, 35(7): 731-736.

[7] Lee YS, Kim SW, Kim SW, et al. Extent of routine central lymph node dissection with small papillary thyroid carcinoma[J]. World J Surg. 2007, 31(10): 1954-1959.
[8] Ito Y, Tomoda C, Uruno T, et al. Clinical significance of metastasis to the central compartment from papillary microcarcinoma of the thyroid[J]. World J Surg, 2006, 30(1): 91-99.

[9] Pereira JA, Jimeno J, Miquel J, et al. Nodal yield, morbidity, and recurrence after central neck dissection for papillary thyroid carcinoma[J]. Surgery. 2005, 138(6): 1095-1101.
[10] Goropoulos A, Karamoshos K, Christodoulou A, et al. Value of the cervical compartments in the surgical treatment of papillary thyroid carcinoma[J]. World J Surg, 2004, 28(12): 1275-1281.
[11] Cheah KW, Arici C, Ituarte PHG, et al. Complications of neck dissections for thyroid cancer[J]. World J Surg. 2002, 26(8): 1013-1016.
[12] Giordano D, Valcavi R, Thompson GB, et al. Complications of central neck dissection in patients with papillary thyroid carcinoma: results of a study on 1087 patients and review of the literature[J]. Thyroid. 2012, 22(9): 911-917.

[13] Sywak M, Cornford L, Roach P, et al. Routine ipsilateral level VI lymphadenectomy reduces postoperative thyroglobulin levels in papillary thyroid cancer[J]. Surgery, 2006, 140(6): 1000-1007.

[14] Dudley NE. Methylene blue for rapid identification of the parathyroids[J]. Br Med J. 1971, 3(5776): 680-681.

[15] Patel HP, Chadwick DR, Harrison BJ, et al. Systematic review of intravenous methylene blue in parathyroid surgery[J]. Br J Surg. 2012, 99(10): 1345-1351.

[16] van der Vorst JR, Schaafsma BE, Verbeek FP, et al. Intraoperative near-infrared fluorescence imaging of parathyroid adenomas with use of low-dose methylene blue[J]. Head Neck. 2014, 36(6): 853-858.

[17] Tummers QR, Schepers A, Hamming JF, et al. Intraoperative guidance in parathyroid surgery using near-infrared fluorescence imaging and low-dose Methylene Blue[J]. Surgery. 2015, 158(5): 1323-1330.

[18] Hillary SL, Guillermet S, Brown NJ, et al. Use of methylene blue and near-infrared fluorescence in thyroid and parathyroid surgery[J]. Langenbecks Arch Surg. 2018, 403(1): 111-118.

[19] Yang F, Jin C, Yang D, et al. Magnetic functionalised carbonnanotubes as drug vehicles for cancer lymph node metastasis treatment[J]. Eur J Cancer. 2011, 47(12): 1873-1882.

[20] Hagiwara A, Takahashi T, Sawai K, et al. Lymph nodal vital staining with newer carbon particle suspensions compared with India ink: experimental and clinical observations[J]. Lymphology. 1992, 25(2): 84-89.

[21] Cheng X, Feng H, Chen L, et al. Intraoperative carbon nanoparticles mapping in secondary total thyroidectomy for recurrent thyroid nodules: Results of a 8-criterion case-match study (case control study)[J]. Int J Surg. 2018, 60: 210-215.

[22] Wang B, Su AP, Xing TF, et al. The function of carbon nanoparticles to improve lymph node dissection and identification of parathyroid glands during thyroid reoperation for carcinoma[J]. Medicine (Baltimore). 2018, 97(32): e11778.

[23] Xue S, Ren P, Wang P, et al. Short and Long-Term Potential Role of Carbon Nanoparticles in Total Thyroidectomy with Central Lymph Node Dissection[J]. Sci Rep. 2018, 8(1): 11936.

[24] Yan S, Zhao W, Wang B, et al. Preoperative injection of carbon nanoparticles is beneficial to the patients with thyroid papillary carcinoma: From a prospective study of 102 cases[J]. Medicine (Baltimore). 2018, 97(27): e11364.

**Study goals and objectives**

### Randomized clinical trials were conducted to further compare the advantages and disadvantages of intraoperative superselective vascular injection methylene blue imaging with traditional parathyroid imaging in parathyroid exploration and postoperative avoidance of hypoparathyroidism, providing clinical basis for further promotion of this method.

### **Study design**

### A total of 300 patients with differentiated thyroid cancer admitted to the Sixth Medical Center of the PLA General Hospital in March 2020 were randomly divided into three groups, with 100 cases in each group. They were divided into group A: The experimental group received ultra-selective vascular methylene blue injection imaging; Group B positive control group: negative development of nano carbon injection; Group C negative control group: intraoperative area imaging was not performed.

### Inclusion and exclusion criteria:

Inclusion criteria: ① differentiated thyroid cancer was confirmed by preoperative FNAC (fine needle puncture pathology) or intraoperative freezing pathology; ② According to the ATA (American thyroid society) standard, for only exists in the unilateral glands, the largest nodule diameter less than 10mm (intraoperative frozen pathology clear) of papillary carcinoma defined as tiny papillary carcinoma, unilateral gland lobe plus gorge resection, unilateral central area lymph node dissection, for the rest of the differentiated cancer with total thyroidectomy, bilateral central area lymph node dissection.

Exclusion criteria: ① age less than 15 years old or more than 75 years old; ② pregnant women; ③ there are serious organic diseases need to complete surgery as soon as possible; ④ History of surgery in the neck area; ⑤ in line with the diagnosis of retrosternal thyroid; ⑥ FNAC or intraoperative pathologic consideration of medullary carcinoma or undifferentiated carcinoma.

**Methodology**

**Intervening measure**

Groupe A: Before the unilateral thyroid gland lobe was removed, the main trunk of the superior thyroid artery was dissected to enter the thyroid gland, and the blood vessels were ligation with 4-0 thin threads. The blood vessels were dissected to the rear of the common carotid artery on the same side in the direction of reverse blood flow, and the superior thyroid artery was clipped at the intersection of the common carotid artery with microvessels to control the proximal and distal blood flow. The blood vessel wall was cut transversally with ophthalmic Venus scissors, and the hose was placed into the artery in the direction of reverse blood flow. 20mg/2ml methylene blue was diluted into 5mg/2ml with normal saline and injected into the artery slowly (2min) through a hose. After the lateral glandular lobe was completely removed (avoiding the removal of the parachromatic gland), the development of the dorsal thyroid methylene blue was observed again. The level of parathyroid hormone (PTH) in the predicted parathyroid tissue was rapidly determined by the immune colloidal gold technique (ICGT) to determine whether the prediction was correct. For bilateral lobectomy, contralateral imaging and parathyroid identification were performed in the same way.

Groupe B: The surface of unilateral thyroid gland lobe was fully exposed, carbon nanoparticle suspension injection was extracted with a 1 ml skin test syringe and slowly injected 0.2 mL around tumor tissue (upper and lower), and withdrawn before injection to avoid blood vessel injection. The level of parathyroid hormone (PTH) in the predicted parathyroid tissue was also rapidly determined by the immune colloidal gold technique (ICGT) after complete resection of the lateral lobe to determine whether the prediction was correct. For bilateral lobectomy, contralateral negative imaging and parathyroid identification were performed with the same method.

Groupe C: Unilateral or bilateral lobotomy is routinely performed. Attention should be paid to the protection of the parathyroid gland according to experience and surgical skills. After complete resection of one side of the gland lobe, the immune colloidal gold technique(ICGT) to determine the level of parathyroid hormone (PTH) in the naked parathyroid tissue to determine whether the prediction is correct; The contralateral parathyroid was determined by the same method.

**Observation indicators and follow-up plan:**

1. The number of correct parathyroid judgments and the total judgments of Groupe A/B/C were counted;

2. For patients with bilateral thyroidectomy and isthmus and central lymph node dissection, intravenous blood sampling was performed to check parathyroid level and blood calcium level before and on the first morning after surgery;

Follow-up plan: (1 follow-up for all enrolled patients 6 months after surgery)

1. If the level of parathyroid hormone in the blood was reviewed 6 months after surgery, if it was less than 50% of the preoperative value, and the patient still had hypocalcemia symptoms such as numbness, tingling, and hand-foot convulsion, it was defined as permanent hypoparathyroidism. The number of patients in Groupe A/B/C group meeting the diagnostic criteria for permanent hypoparathyroidism was counted.

Among them, 1 and 3 are the main evaluation indicators, and 2 are the secondary evaluation indicators.

**Flow diagram**


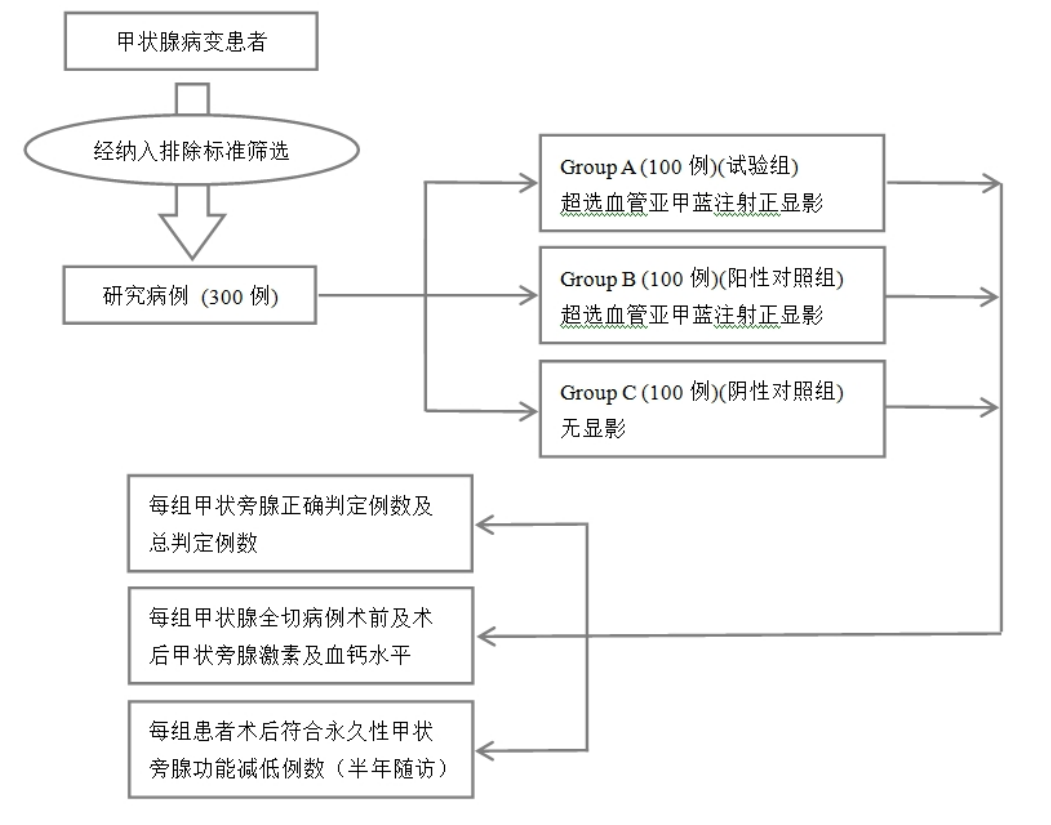


### **Data management**

### Subject information will be collected through paper CRF. Data entry and management Using Epidata software, two people two copies of independent data entry. Check and correct the missing values, outliers and logical errors of the data, and lock the database after the data is cleaned.

**Statistical analysis**

SPSS 25.0 software was used for statistical analysis. Mean and standard deviation were used for statistical description of quantitative indicators, and one-way ANOVA, Kruskal-Wallis rank sum test or ANOVA of repeated measurement data were used for hypothesis testing. The statistical description of the qualitative indicators used the case number, rate or composition ratio, and the Chi-square test or Fisher exact probability method were used for comparison between groups. Test level α=0.05.

### **Ethics**

The operation involved in this study, "unilateral or bilateral thyroidectomy + central lymph node dissection", is the current standard radical treatment for differentiated thyroid cancer. Negative carbon nanography (Group B) has been promoted in China for nearly 10 years, and has been carried out in a large number of top three hospitals, and its safety and effectiveness have been well verified. Therefore, the setting of Group B in this subject does not violate medical ethics.

At present, the recommendations for intraoperative carbon nano injection are mainly found in Chinese guidelines: the Expert Consensus on parathyroid Protection during thyroid Surgery (2015 edition) recommended grade A, and the recommendation grade of the Guidelines for Perioperative Thyroid Parathyroid Protection (2018 edition) dropped to grade B, while the recommendations in international guidelines (including ATA, ETA, and AOTA) were reduced to grade B. There is no content related to carbon nanoparticles in NCCN and other thyroid related guidelines. In the current clinical practice of major hospitals in China, carbon nanoparticles injection is only an optional method, rather than a necessary means. Therefore, the setting of Group C in this subject does not violate medical ethics. For Group C, we will try our best to identify the parathyroid gland and avoid its injury through careful dissection and surgical experience.

Group A involves intravascular injection of methylene blue. According to the instructions for methylene blue injection, excessive intravenous injection (0.5g) can cause headache, dizziness, precardiac pain, sweating, confusion, and low or inverted T-wave. In this study, only 5mg or 10mg was injected in one patient at a time (depending on whether the operation was unilateral or bilateral resection), and the dose of methylene blue into the systemic circulation was reduced by temporarily blocking the proximal inferior thyroid artery, while part of methylene blue was removed immediately after entering the thyroid gland, which may cause less adverse reactions. In addition, the puncture point of methylene blue injection was located at the distal end of the blood supply vessel of the parathyroid gland. Theoretically, ligation of the puncture point after injection of developer would not affect the blood supply of the parathyroid gland. Therefore, the setting of Group A in this subject does not violate medical ethics.
